# Hotspots in research on the measurement of medical students’ clinical competence from 2012-2016 based on co-word analysis

**DOI:** 10.1186/s12909-017-0999-8

**Published:** 2017-09-12

**Authors:** Xing Chang, Xin Zhou, Linzhi Luo, Chengjia Yang, Hui Pan, Shuyang Zhang

**Affiliations:** 1 Department of Education, Peking Union Medical College Hospital, Chinese Academy of Medical Sciences & Peking Union Medical College, No.1 Shuaifuyuan Wangfujing, Dongcheng District, Beijing, 100730 China; 2 Product Management Department, China Export & Credit Insurance Corporation, Beijing, 100032 China; 3 Department of Cardiology, Peking Union Medical College Hospital, Chinese Academy of Medical Sciences & Peking Union Medical College, No.1 Shuaifuyuan Wangfujing, Dongcheng District, Beijing, 100730 China

**Keywords:** Measurement of clinical competence, Hotspots, Co-word analysis, Medical students

## Abstract

**Background:**

This study aimed to identify hotspots in research on clinical competence measurements from 2012 to 2016.

**Methods:**

The authors retrieved literature published between 2012 and 2016 from PubMed using selected medical subject headings (MeSH) terms. They used BibExcel software to generate high-frequency MeSH terms and identified hotspots by co-word analysis and cluster analysis.

**Results:**

The authors searched 588 related articles and identified 31 high-frequency MeSH terms. In addition, they obtained 6 groups of high-frequency MeSH terms that reflected the domain hotspots.

**Conclusions:**

This study identified 6 hotspots of domain research, including studies on influencing factors and perception evaluation, improving and developing measurement tools, feedback measurement, measurement approaches based on computer simulation, the measurement of specific students in different learning phases, and the measurement of students’ communication ability. All of these research topics could provide useful information for educators and researchers to continually conduct in-depth studies.

## Background

For medical students, as future doctors, clinical competence is one of the most important capabilities to acquire. However, the measure used to evaluate this capability remains a key point of concern, and this study domain is currently a major theme in the field of medical education [1–3]. Moreover, research on the measurement of medical students’ clinical competence covers many different topics and themes, including the testing of measurement approaches, the measurement of a clerk’s clinical ability and students’ perceptions of clinical competence evaluations [4].

Although previous systematic reviews have summarized the progress in research regarding the measurement of clinical competence, researchers have paid more attention to one or a few aspects of this study domain [5–7]. Therefore, the published literature lacks a comprehensive summary of the research on the measurement of medical students’ clinical competence. We sought to determine which research themes were the most frequently published, i.e., research hotspots, with the aim of facilitating future studies and education.

As a common approach to bibliometrics, co-word analysis was first developed by French [8]. Co-word analysis is a type of quantitative analysis that reflects the content of publications by analyzing the frequencies and relationships of co-words. Its main principles are the following: keywords are used to reflect the main content of articles. If two keywords expressing particular research subjects appear in the same article, there may be a certain intrinsic relationship between the two keywords. The more frequently the two keywords occur in the same publications, the closer is the relationship between the words and the more popular are the research subjects reflected by the two keywords. According to the frequency of co-words, keywords can be classified into groups by statistical methods, such as cluster analysis or factor analysis. Additionally, different keywords groups represent particular research hotspots. Currently, this method is widely applied to map the knowledge structure of research fields [9], identify research domain topics, and explore the characteristics and development of the evolution of specific subjects. In addition, this type of analysis has been used in many fields, such as medical science, environmental science, and biology.

Accordingly, using a co-word analysis of the existing literature, we aimed to identify hot topics in the research on the measurement of medical students’ clinical competence and to determine crucial sub-domains of this field of study to provide useful evidence for medical educators and researchers.

## Methods

### Data source

As a professional database, PubMed contains the world’s largest body of literature on biomedicine. In addition, PubMed has created medical subject heading (MeSH) terms. PubMed indexes most of the literature with MeSH terms, which reflect the contents of the literature more accurately than keywords. Generally, the results derived from a co-word analysis based on MeSH terms are more reliable and reasonable than those obtained using keywords. Therefore, we chose PubMed as the data source for this study.

When we searched the literature, we performed a MeSH search due to its high accuracy. The retrieval strategy was as follows: ((((((((Clinical skill) OR Clinical capacity) OR Clinical performance) OR Clinical ability)) OR “Clinical Competence”[Mesh])) AND “Students, Medical”[Mesh]) AND ((((education* assess*) OR education* evaluat*)) OR “Educational Measurement”[Mesh:NoExp]). Moreover, we set the publication date from 2012/01/01 to 2016/12/31, and we performed the process of retrieval on January 6, 2017.

### Criteria for literature selection

We selected literature that met the following study criteria: [1] the type of study was an original article, and [2] the articles searched were related to the topic of “measurement of medical students’ clinical competence.” We excluded literature if any of the following were true: [1] the study type was not an article, i.e., it was a review or a letter, and [2] the main contents of the study did not concern the measurement of medical students’ clinical competence.

We exported the literature results from PubMed in a Medline file. Then, two researchers independently checked the literature by title, abstract and the full text if needed. Finally, we obtained a new Medline file that included the articles that met the criteria of our study.

### Data analysis

We deleted specific terms representing characteristics of demography and geographic location, such as “infants” and “Asia”, that were not related to this research. We also extracted the remaining MeSH terms from the new Medline file. We then created a new TXT file in which the MeSH terms were arranged according to different articles. Next, we imported the TXT file into the BibExcel software (developed by Olle Persson), which counted the frequency of each MeSH term. Then, we identified high-frequency MeSH terms based on the Donohue formula, $T=\frac{-1+\sqrt{1+8\times \mathrm{I}1}}{2}$, where *I*_*1*_ represents the number of MeSH terms indexed only once in the articles. If the frequency of a MeSH term was greater than the value of *T*, that term was considered a high-frequency MeSH term. We used these high-frequency MeSH terms to generate a co-word matrix using BibExcel. Furthermore, we transformed the co-word matrix into a correlation matrix by calculating the Ochiai coefficient using the following formula: $\mathrm{Ochiai}=\frac{N}{\sqrt{Na\times Nb}}$. In this formula, *N* represents the frequency at which term A and term B were both indexed in the same articles; *Na* represents the frequency of term A; and *Nb* represents the frequency of term B. Finally, we imported the correlation matrix into SPSS version 18.0 for Windows (SPSS Inc., Chicago, Illinois) to perform a cluster analysis. We obtained a co-word cluster diagram of the high-frequency MeSH terms, which indicated the research hotspots.

## Results

### Selected literature

**Fig. 1:**
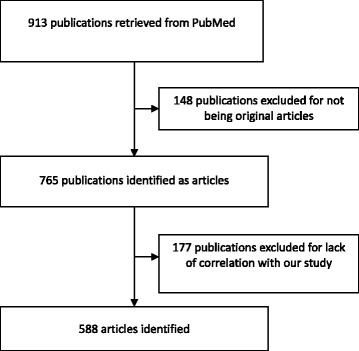
Flow diagram illustrating the literature search

As shown in Fig. 1, 913 studies were retrieved from PubMed, of which 588 met the literature selection criteria and were extracted in this research.

### High-frequency MeSH terms

From the 588 original articles, 833 MeSH terms were indexed, and the total occurrence of MeSH terms was 5625. In other words, each MeSH term appeared an average of 6.75 times in the retrieved articles. According to the Donohue formula, the value of *T* equaled nearly 29, which identified MeSH terms with a frequency greater than 29 as high-frequency words. Ultimately, 31 high-frequency MeSH terms were extracted, as shown in Table 1. “Students, Medical” (508, 9.03%), “Clinical Competence” (438, 7.79%), and “Educational Measurement” (362, 6.44%) were the top three MeSH terms.

**Table 1:** Descriptions of the high-frequency MeSH terms

NO.	MeSH term	Total frequency of the term	Percent frequency
1	Students, Medical	508	9.03%
2	Clinical Competence	438	7.79%
3	Educational Measurement	362	6.44%
4	Education, Medical, Undergraduate	270	4.80%
5	Curriculum	168	2.99%
6	Surveys and Questionnaires	117	2.08%
7	Clinical Clerkship	97	1.72%
8	Education, Medical	90	1.60%
9	Program Evaluation	68	1.21%
10	Attitude of Health Personnel	67	1.19%
11	Teaching	65	1.16%
12	Learning	64	1.14%
13	Reproducibility of Results	63	1.12%
14	Internship and Residency	61	1.08%
15	Communication	60	1.07%
16	Schools, Medical	59	1.05%
17	Patient Simulation	51	0.91%
18	Cross-Sectional Studies	48	0.85%
19	Faculty, Medical	46	0.82%
20	Physician-Patient Relations	44	0.78%
21	Prospective Studies	40	0.71%
22	Problem-Based Learning	39	0.69%
23	Physical Examination	37	0.66%
24	Computer Simulation	37	0.66%
25	Health Knowledge, Attitudes, Practice	36	0.64%
26	Education, Medical, Graduate	33	0.59%
27	Feedback	32	0.57%
28	Retrospective Studies	32	0.57%
29	General Surgery	31	0.55%
30	Pilot Projects	31	0.55%

The 31 high-frequency MeSH terms mainly showed five layers of clinical competence measurement: measurement subjects (2 terms), measurement objects (5 terms), measurement contents (5 terms), measurement methods or tools (4 terms), and activities of medical education (7 terms), which were shown in Table 1. The first four layers were associated with clinical competence measurement, as they were the key parts of the measurement process. Although there was no direct correlation between the fifth layer and clinical competence measurement, medical educational activities had an important effect on the measurement.

### Co-word cluster of high-frequency MeSH terms

**Fig. 2:**
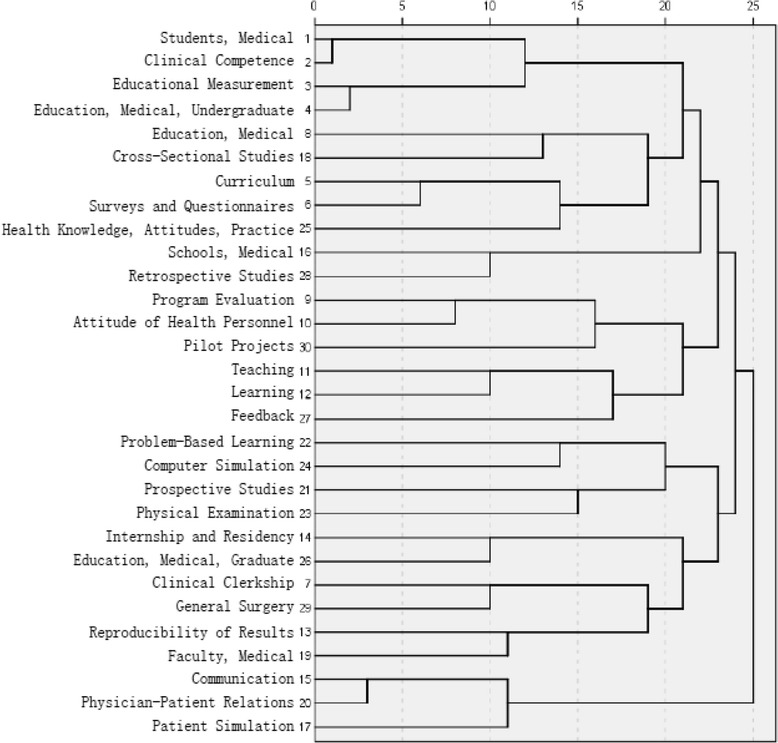
Cluster diagram of high-frequency MeSH terms

According to the frequency of co-words, high-frequency MeSH terms were subjected to a cluster analysis; the results are shown in Fig. 2. According to the distance of the cluster, which equaled 22, we divided 31 MeSH terms into 6 groups, which revealed hotspots for this study domain.

The first group contained the most MeSH terms [11], which included terms of measurement objects (“students, medical” and “education, medical, undergraduate”), measurement content (“clinical competence” and “health knowledge, attitudes, practice”), and activities of medical education (“education, medical” and “curriculum”). These terms occurred in the same publication, implying that there must be a link among students, clinical competence and curriculums. To our knowledge, as an essential influencing factor, whether experienced curriculums would cause different results. Hence, researchers took factors associated with curriculums into account [10, 11] when determining the connection between curriculums and the results of clinical competence assessments.

The second group included terms of measurement methods (“program evaluation”) and measurement contents (“attitude of health personnel”). These terms simultaneously appeared in publications with the term “pilot projects”, which revealed the new measurement methods that were explored and implemented by medical educators [12], who tested the effects, validity and feasibility of new methods [13]. The third co-word cluster contained terms of medical education activities (“teaching”, “learning”, and “feedback”). These three MeSH terms occurred simultaneously in articles regarding clinical competence measurement, indicating that educators and students were focused on this measurement. The role of feedback was realized, and its benefit guided future clinical training and evaluation. Thus, in this field, researchers mainly studied teachers’ and students’ perceptions and satisfaction [14, 15] to improve measurements of education.

The fourth co-word cluster contained terms of measurement methods (“physical examination”) and educational activities (“problem-based learning” and “computer simulation”). Computer simulation was gradually applied to the processes of learning and teaching. Thus, a question explored by researchers was whether simulation training would improve clinical skills [16] and whether the effectiveness of training with computer simulation was statistically significant.

The fifth co-word cluster result contained terms of measurement subjects (“Faculty, Medical”) and measurement objects (“internship and residency”, “education, medical, graduate” and “clinical clerkship”). The four high-frequency terms of co-occurrence reflected that medical educators paid more attention to the important steps in the process of learning, and they wanted to determine students’ clinical performance in these phrases [17, 18].

The sixth co-word cluster contained terms of measurement contents (“communication”, “physician-patient relations”) and methods (“patient simulation”). These three high-frequency terms occurred simultaneously in the articles, revealing that researchers were interested in the relationship between patients and medical students, especially the assessment of communication skills by patient simulation [19]. From the above results, six hotspots of clinical competence measurement were found, as follows: [1] the impact of curriculums on clinical competence, [2] development of new methods of clinical competence measurement, [3] students’ feedback on clinical competence measurement, [4] the effect of computer simulation training on clinical competence, [5] students’ clinical performance in the important steps of learning, and [6] communication skills measured by patient simulation.

## Discussion

Potential evidence of findings.

According to the co-word analysis, we identified 6 hotspots of medical students’ clinical competence measurement. In light of these 6 hot topics, we found several potential features.

[1] Clinical competence has a broader goal: knowledge, skills and attitudes. Because students’ level of training varies, there are great differences among the measurements in different steps. Junior students are tested by examinations to determine whether they have acquired knowledge, while the comprehensive capabilities of resident doctors are assessed by patient simulation. Hence, it is important for educators to choose appropriate measurement objectives and methods and study the feasibility of each method.

[2] Advanced technology would be useful to medical education and would overcome the deficiencies of traditional measurement tools. The combination of educational and technical assessments will become a trend in the future. With a myriad of innovations in the measurement process, researchers should not only pay attention to their validity and reliability but also start concentrating on creating standards. Ultimately, “pilot projects” will be applied worldwide to promote medical education.

[3] Training is a crucial factor that affects clinical competence. There are multiple forms of training, including curriculums, bedside teaching, and case discussions. However, researchers paid much more attention to curriculums than to other training forms. The reason for this finding might be that curriculums are the major form of training, and it is easy to identify the impact of curricula on clinical competence, such as by examination. In fact, the level of clinical competence is the result of many factors. Accordingly, researchers need to study further how to measure different impact factors and how to improve students’ clinical competence.

### Applications of co-word analysis

In light of the increasing importance of clinical competence measurement over the past decades, we decided to identify hot topics in research in this domain using a co-word analysis. Our study represented the first detailed analysis of global clinical competence measurement research hotspots from 2012 to 2016.

Methodologically, hotspots provide useful information for educators and researchers. For educators, understanding hot research domains allows them to consider clinical competence measurement research in budgets. They can also discuss whether to adopt a new measurement tool to assess students’ clinical competence. For researchers, hotspots could provide evidence of research theme selection to facilitate their future research. Moreover, researchers could receive guidance in the search for new research areas and conduct further studies on the basis of their findings. For instance, they could study the conditions and range of applications of new measurement methods.

Scientific findings, such as publications, contain vast amounts of information and clues regarding research topics. We can use this information to study research activity. As a representation of publication contents, keywords or MeSH terms could be used to explore the characteristics of research. However, there are some guidelines for this research method. First, when a co-word analysis is used for research, a large number of high-frequency co-words are needed. Therefore, a minimum number of published articles is required, at least a few hundred. Second, the relationship between co-words must be reflected by statistical software (such as SPSS) or bibliometric tools (for example, CiteSpace and VOSviewer). In particular, with the rapid development of knowledge visualization, potential information from publications could be visually demonstrated using figures. Third, co-word analysis could be used to study the structure and evolution of research in a particular field [20] and to explore new research subjects in addition to identifying hotspots.

Several limitations of our study should be noted. MeSH terms are standard words used to index studies, although not all words have related MeSH terms, especially emerging words; this discrepancy may have affected the results of the co-word analysis to some extent. We also did not divide the MeSH terms by calendar year; therefore, we could not explore the annual evolution of changes in research hotspots.

## Conclusions

Clinical competence measurement is systematic and sophisticated. We found six research hotspots referring to aspects of the measurement process, and these findings should be helpful for educators and researchers.

## Abbreviations

MeSH: Medical subject headings
